# Producing and Characterizing Polyhydroxyalkanoates from Starch and Chickpea Waste Using Mixed Microbial Cultures in Solid-State Fermentation

**DOI:** 10.3390/polym16233407

**Published:** 2024-12-03

**Authors:** Karlo Grgurević, Dora Bramberger, Martina Miloloža, Krešimir Stublić, Vesna Ocelić Bulatović, Jasmina Ranilović, Šime Ukić, Tomislav Bolanča, Matija Cvetnić, Marinko Markić, Dajana Kučić Grgić

**Affiliations:** 1 Faculty of Chemical Engineering and Technology, University of Zagreb, Marulićev trg 19, 10000 Zagreb, Croatia; kgrgurevi@fkit.hr (K.G.); dbramberg@fkit.hr (D.B.); miloloza@fkit.hr (M.M.); vocelicbulatovic@fkit.unizg.hr (V.O.B.); sukic@fkit.unizg.hr (Š.U.); tbolanca@fkit.unizg.hr (T.B.); mcvetnic@fkit.unizg.hr (M.C.); mmarkic@fkit.unizg.hr (M.M.); 2 Aqua V.M.V. d.o.o., Kralja Zvonimira 98, 10000 Zagreb, Croatia; 3 Podravka Inc., Ante Starčevića 32, 48000 Koprivnica, Croatia; jasmina.ranilovic@podravka.hr

**Keywords:** agro-industrial waste, isolation and identification, solid-state fermentation, polyhydroxyalkanoates

## Abstract

The environmental impact of plastic waste is a growing global challenge, primarily due to non-biodegradable plastics from fossil resources that accumulate in ecosystems. Biodegradable polymers like polyhydroxyalkanoates (PHAs) offer a sustainable alternative. PHAs are microbial biopolymers produced by microorganisms using renewable substrates, including agro-industrial byproducts, making them eco-friendly and cost-effective. This study focused on the isolation and characterization of PHA-producing microorganisms from agro-industrial waste, including chickpeas, chickpeas with bean residues, and starch. Screening via Sudan Black staining identified PHA-accumulating strains such as *Brevibacillus* sp., *Micrococcus* spp., and *Candida krusei*, among others. To assess the potential for PHA biosynthesis, solid-state fermentation (SSF) was conducted using agro-industrial waste as substrates, along with a mixed culture of the isolated microorganisms. The highest observed yield was a PHA accumulation of 13.81%, achieved with chickpeas containing bean residues. Structural and thermal characterization of the PHAs was performed using Fourier-transform infrared spectroscopy with attenuated total reflectance (FTIR-ATR), differential scanning calorimetry (DSC), and thermogravimetric analysis (TGA). FTIR-ATR spectra indicated polyhydroxybutyrate (PHB), suggesting it as the synthesized PHA type. This study highlights the potential of agro-industrial waste for sustainable PHA production and eco-friendly bioplastics.

## 1. Introduction

Plastics obtained from oil and its derivatives make one of the widely used materials in various aspects of human life. However, due to their increasing demand and production, new problems are arising such as plastics’ toxicity, adequate disposal of plastics, and their excessive accumulation in the environment [1]. According to Sabapathy et al. [2], over 4.8 to 12.7 million tons of plastics end up disposed in world’s oceans. Synthetic polymers exhibit low biodegradability, making recycling alone an inadequate solution for managing their environmental impact. Consequently, scientific research must prioritize the development of new materials that combine high biodegradability with the mechanical and thermal properties characteristic of synthetic polymers, aiming to replace them effectively [1]. In this context, bioplastics have become a major focus of study. Given the significant advancements already made in this field, bioplastics are now broadly classified into natural and synthetic biopolymers. Natural biopolymers are further categorized based on their production sources: those derived from biomass resources (such as polypeptides, lipids, and polysaccharides), those produced through biotechnological processes (e.g., polylactide, PLA), and those synthesized by microorganisms (such as polyhydroxyalkanoates, PHA). Synthetic biopolymers can likewise be categorized into three main groups: aliphatic biopolyesters (such as polycaprolactone, PCL), aliphatic copolyesters (such as polybutylene succinate, PBS), and aromatic copolyesters (such as polybutylene adipate terephthalate, PBAT) [3]. According to data from European Bioplastics [4], global plastic production exceeds 400 million tons annually, of which only around 0.5% consists of bioplastics. In 2023, the global production capacity for bioplastics was approximately 2.18 million tons. Of this, biodegradable plastics represented 52.1%, with PLA accounting for 31.0%, starch-containing polymer compounds (SCPC) for 6.4%, and PHAs for 4.8%, making them the most widely produced types. Production capacity for bioplastics is projected to grow to 7.43 million tons by the end of 2028, with biodegradable bioplastics expected to constitute 62.0% of this capacity. In this paper, PHAs and their synthesis methods will be further discussed.

PHAs are defined as biodegradable polyesters composed of various hydroxyalkanoate monomers [1]. This material has several properties, like high crystallinity, biodegradability, and resistance against ultraviolet radiation. Due to the presence of these properties, this material has been used for the packaging material of foods and medicines [5]. PHA are notable for their unique biosynthesis process, which occurs under nutrient-limited conditions in specific microorganisms. In these conditions, a substrate rich in carbon but limited in nitrogen, phosphorus, magnesium, and other essential nutrients is provided. Under such fermentation conditions, microorganisms accumulate different types of PHAs as an alternative energy storage compound [6]. Currently, over 150 different types of PHAs have been identified, primarily classified by the number of carbon atoms in the side chain of their monomer units. PHAs are categorized into three main groups: short-chain-length PHAs (scl-PHAs), which contain 3 to 5 carbon atoms; medium-chain-length PHAs (mcl-PHAs), which consist of 6 to 14 carbon atoms; and long-chain-length PHAs (lcl-PHAs), which have 15 or more carbon atoms in their side chains [7]. Additionally, PHAs can be classified as either homopolymers (homopolyesters), composed of a single type of monomer, or copolymers (copolyesters), made up of two or more different monomers [8]. Among the most produced PHAs by microorganisms are poly(3-hydroxybutyrate) (PHB) and poly(3-hydroxybutyrate-co-3-hydroxyvalerate) (PHBV). PHB belongs to the scl-PHA group, while PHBV is categorized within the scl- and mcl-PHA copolymer group [9,10]. PHBV is the most influential and inclusive biodegradable polymer among the PHA polymer family, showcasing significant potential as a sustainable replacement for conventional plastics [5]. Microorganisms capable of producing scl-PHAs include *Bacillus megaterium* [11], *Cupriavidus necator* [12,13], *Escherichia coli* [14], *Bacillus cereus* [15], *Chromobacterium violaceum* [16,17], *Alcaligenes latus* [18], *Paracoccus denitrificans* [19], *Azotobacter vinelandii* [20], *Halomonas alkalicola* [21], and *Bacillus megaterium* [22]; mcl-PHA producers include *Aeromonas hydrophila* [23], *Pseudomonas putida* [24,25], *Pseudomonas nitroreducens* [26,27], *Pseudomonas aeruginosa* [25,28], *Pseudomonas entomophila* [29]; and lcl-PHA producers include *Pseudomonas aeruginosa* [30,31] and *Bacillus thuringiensis* [32].

One of the most common methods for producing PHAs is through the fermentation of sugars and fatty acids [33]. There are two generations of biomass that can be utilized in this fermentation process. The first generation consists of food products that serve as substrates for PHA production, including corn, sugarcane, beans, and nuts. While these resources are widely available, utilizing food products for PHA production can lead to increased food prices and a reduction in food supply. According to the Food and Agriculture Organization of the United Nations (FAO) [34], replacing the 170 million tons of global plastic food packaging produced annually with bioplastics would require 54% of corn crops and over 60% of Europe’s drinking water. Consequently, alternative sources such as agro-industrial waste are increasingly utilized as feedstock for PHA production [35,36]. This second generation of biomass, characterized by its lignocellulosic structure, generates over 1.3 billion tons annually [37], with much of it being disposed of in landfills or incinerated. This waste contributes significantly to environmental issues, including greenhouse gas emissions and toxic degradation byproducts, resulting in a carbon footprint equivalent to approximately 3.3 billion tons of CO_2_ released into the atmosphere. These facts highlight the urgent need to address agro-industrial waste through alternative methods that align with the principles of a circular economy.

Potato starch waste and chickpea waste are among the promising agro-industrial residues for PHA production. Potato starch waste is a by-product of industrial potato processing, primarily recovered from wastewater used to rinse peeled potatoes, along with other starch sources such as residual tubers, peels, and potato pulp. After sedimentation and starch separation, the remaining starch is filtered and dried [38,39,40]. Starch, comprising the polysaccharides amylose and amylopectin, is an abundant source of glucose monomers, which makes it a highly suitable substrate for PHA synthesis [41]. Chickpea waste, derived from the legume *Cicer arietinum*, is rich in macromolecules, including proteins [12.4–31.5%], starch [41–50%], unsaturated fatty acids (6%), lignocellulose, vitamins (e.g., riboflavin, thiamine), and minerals (iron, calcium) [42,43]. Primarily used as a protein source, chickpeas undergo processing that removes husks, leaving behind husks and spoiled chickpeas as waste. With suitable pretreatment, these macromolecules can be broken down into components usable by microorganisms for PHA synthesis [44].

Lignocellulose is composed of three main components: cellulose, hemicellulose, and lignin. Cellulose and hemicellulose form an interconnected fibrous network, with lignin providing structural reinforcement. A significant challenge in utilizing agro-industrial waste for PHA production lies in the inherent resistance of lignocellulosic materials to microbial degradation. To enhance their bioavailability, various pretreatment methods are employed, including physical (e.g., ultrasonic [45], thermal [46,47,48], hydrothermal [49,50]), chemical (e.g., acidic hydrolysis [51,52], alkaline hydrolysis [53,54,55], oxidative agents [56]), physico-chemical (e.g., steam explosion [57,58,59], combined ultrasonic and alkaline treatments [60,61,62], extrusion [63]), biological (e.g., bacterial [64], fungal [65], enzymatic [66,67,68,69]), and “green-solvent” methods (e.g., ionic liquids [70], eutectic solvents [71,72], supercritical fluids [73]). These pretreatment strategies enhance waste porosity, surface area, and particle size optimization, increasing the concentration of reducing sugars essential for fermentation [35,74,75]. Table 1 summarizes agro-industrial wastes, corresponding pretreatment methods, and microbial species utilized for PHA synthesis.

For achieving high levels of PHA accumulation, specific cultivation conditions must be optimized, including moisture, pH, temperature, and the carbon-to-nitrogen (C/N) ratio [82]. Moisture content is essential as it supports fermentation by creating an optimal environment for microbial growth. Insufficient moisture can lower nutrient solubility, while excessive moisture may inhibit enzyme activity. So, the ideal level should align with the biological needs of the microorganisms used [75]. Research by Catherine et al. [83] indicates that, within a certain range, pH has minimal impact on final PHA accumulation. For instance, pH values of 6.8, 7.3, 7.8, 8.3, and 8.8 led to PHA yields between 18 and 20% of dry cell weight, showing negligible variation. Conversely, Villano et al. [84] observed a decline in PHA yield as pH rose from 7.5 to 9.5, suggesting that the effect of pH on PHA accumulation may depend on the substrate, microorganisms, or other experimental parameters. Temperature also plays a critical role, with the highest PHA accumulations reported between 20 °C and 35 °C [83,85]. Temperatures outside this range can partially inactivate or inhibit metabolic pathways, reducing PHA production or even leading to PHA degradation. The C/N ratio further influences PHA synthesis, though requirements may vary by microorganism. Generally, the best yields are achieved with a high C/N ratio, as PHA production is favored in conditions where carbon is abundant, and nitrogen, magnesium, and phosphorus are limited. Wang et al. [86] demonstrated this by testing glucose and ammonium chloride in C/N ratios of 3.6:1, 36:1, and 360:1. The results confirmed that the highest PHA accumulation occurred at a 360:1 ratio, underscoring the importance of a high C/N ratio for optimal PHA yield.

PHA production can be achieved through two primary fermentation methods: Solid-State Fermentation (SSF) and Submerged Fermentation (SmF). In the SSF process, microorganisms grow and produce PHAs using a solid substrate that provides both support and nutrients. The substrate must contain an optimal level of moisture to facilitate microbial metabolic activity. SSF is particularly valuable for bioconversion of agro-industrial wastes into biofuels, biomaterials, or chemicals. The process can be divided into three stages: upstream, midstream, and downstream processes. During the upstream phase, the substrate and growth media are prepared, and microorganisms (often sourced from the waste itself) are isolated. The midstream phase is centered around fermentation, including substrate inoculation, incubation, and continuous monitoring and control of fermentation conditions. In the downstream phase, the final product is extracted, purified, and any residual biomass and organic waste are disposed of properly [87,88,89]. In contrast, the SmF process utilizes an aqueous system containing various nutrients and microbial suspension. SmF can be carried out in both batch and continuous (flow) bioreactors. In a batch process, nutrients and inoculum are added once, and the process ends when all nutrients have been converted into the desired product. The flow bioreactor, however, operates as an open system, where liquid nutrient broth and inoculum are continuously added while the product is simultaneously removed. As in SSF, any residual biomass from the fermentation is properly disposed of after the process is completed. Throughout both fermentation methods, key parameters such as temperature, pH, oxygen levels, and nutrient concentrations are continuously monitored to ensure optimal microbial growth and PHA production [90,91,92]. 

The following step in PHA production is extraction, which can roughly be divided into three parts. Extraction usually begins with the separation of dry cell weight from the residual biomass. After that, the cell wall is pretreated by heating, freezing, and thawing or by using alkaline or sodium chloride solution. These methods allow the PHA granules to exit the cells easily. The last part includes disrupting the cell completely and genuine extraction of PHAs by using appropriate solvent [8]. Cell disruption is most often carried out with sodium hypochlorite [93,94,95], hydrogen peroxide [96], various surfactants [97,98], enzymes [99] etc. Generally, using sodium hypochlorite, surfactants or enzymes gives high-quality and high-purity products. However, sodium hypochlorite can severely reduce the molecular weight of the obtained PHA, while using surfactants or enzymes is not financially beneficial due to the cost of the enzymes and the need for surfactant wastewater treatment [100]. To reduce the mentioned disadvantages, these chemicals can be combined in the cell disruption process. Marudkla et al. [101] used sodium dodecyl sulfate (0.5% *w*/*v*) and sodium hypochlorite (6% *v*/*v*) for PHB extraction and recorded 78.70% PHB recovery. Since PHAs are highly soluble in chlorinated hydrocarbons (mainly chloroform [102,103], dichloromethane [104]), they are frequently used for their extraction from microorganisms’ cells. Their main disadvantages are their toxicity and a large amount that should be disposed after the extraction [99]. Recently, methods such as supercritical fluid extraction [105,106], eutectic solvent extraction [71] and phage lysis [107] have been investigated. These methods have shown to be highly selective for PHAs, rapid, and environmentally friendly; however they are dependent on strictly defined process parameters that have to be constantly controlled and optimized (phage lysis can be carried out only on genetically modified microorganisms) [8].

The objective of this study was to isolate and identify microorganisms from various agro-industrial waste biomass sources, including chickpeas, chickpea-bean residues, and starch, in order to assess their potential for PHA production through SSF. Furthermore, the study aimed to characterize the produced PHA polymers using FTIR-ATR spectroscopy, differential scanning calorimetry (DSC), and thermogravimetric analysis (TGA). The findings were intended to offer valuable insights into the feasibility of utilizing agro-industrial waste as a sustainable feedstock for biopolymer production, potentially reducing the environmental impact of plastic pollution.

## 2. Materials and Methods

### 2.1. Physico-Chemical Characterization of Agricultural Waste

The substrates used in this experiment included waste starch, waste chickpeas (referred to as chickpea 1), and waste chickpeas with bean residues (referred to as chickpea 2). The starch was obtained from Kanaan d.o.o., located in Donji Miholjac, Croatia, while both types of waste chickpeas were sourced from Podravka d.d. in Koprivnica, Croatia. For the experiments, the substrates were homogenized using a knife mill GRINDOMIX GM 200 (Retsch GmbH, Haan, Germany) to achieve a uniform particle size. The substrates were analyzed for moisture content (MC) [108], dry matter (DM), volatile matter (VM) [109], nitrogen [110], and carbon [111] content. Additionally, the pH value and concentration of reducing sugars (RS) were measured to assess the chemical characteristics of the substrates. The pH-value of the substrates was determined by SenTix^®^ 940 electrode (Xylem Inc., New York, NY, USA) and the concentration of RS was determined using the DNS method [112]. For this analysis, 10 g of each substrate (dry weight) was mixed with 100 mL of distilled water in 250 mL Erlenmeyer flasks and incubated at 37 °C for 40 min. Following incubation, the mixtures were subjected to centrifugation at 5500 rpm for 10 min using a Sigma 3K15 centrifuge (Sigma Laborzentrifugen GmbH, Osterode am Harz, Germany). The supernatant was then decanted, and 1 mL of each sample was transferred into cuvettes. Subsequently, 1.5 mL of DNS reagent was added to each sample, and the absorbance for determining the concentration of reducing sugars was measured at a wavelength of 575 nm using a DR3900 spectrophotometer (Hach, Loveland, CO, USA). These analyses were essential for understanding the physical and chemical properties of the substrates, which are crucial for evaluating their suitability as growth media for microbial isolation and subsequent fermentation processes.

### 2.2. Isolation and Identification of PHA-Producing Microorganisms

To isolate microorganisms from substrates, waste starch, chickpea 1, and chickpea 2, 10 g of each substrate (dry weight) was weighed and suspended in 100 mL of sterile distilled water in Erlenmeyer flasks. Eluates were prepared following ISO 21268-3:2019 [113] guidelines. The flasks were placed on a Biosan PSU-20i Orbital Shaker (Biosan, Riga, Latvia) at 160 rpm for 24 h at ambient temperature. After 24 h, bacterial and fungal colony-forming units (CFUs) were quantified on general-purpose media: nutrient agar (NA) for bacterial colonies and malt agar (MA) for fungal colonies, using the pour plate method as described by Briški et al. [114]. For all substrates, serial dilutions were prepared using a 0.9% NaCl aqueous solution (from 10^0^ to 10^−9^), plated, and colonies were enumerated on the plates where the colony count fell within the optimal range of 20 to 400, ensuring accuracy and statistical reliability in the quantification of microbial populations. The plates were incubated at 80% relative humidity, with fungi cultivated at 28 °C and bacteria at 37 °C. After incubation, the number of colonies on agar plates were determined. The average CFU values for bacteria and fungi were determined by counting two serial dilutions as follows:

Before the experiment: Bacterial CFU: chickpea 1: 10^−8^ and 10^−9^; chickpea 2 and starch: 10^−7^ and 10^−8^; Fungal CFU: chickpea 1 and starch: 10^−5^ and 10^−6^; chickpea 2: 10^−6^ and 10^−7^.

At the end of the experiment: Bacterial CFU: chickpea 1 and 2: 10^−8^ and 10^−9^; starch: 10^−7^ and 10^−8^; Fungal CFU: chickpea 1 and 2; and starch: 10^−6^ and 10^−7^.

The results were expressed as CFU of microorganisms per gram of dry matter. Bacterial and fungal colonies with distinct morphological characteristics and predominance on nutrient agar (NA) and malt agar (MA) plates were selected and transferred to fresh NA and MA plates for isolation. Bacterial isolates were incubated at 37 °C for 24–48 h, while fungal isolates required incubation at 28 °C for 3–5 days, as outlined in the method by Briški et al. [114]. To ensure purity, transfers to fresh media were repeated until single-species colonies were confirmed. After isolating pure cultures, each was preserved on slant agar to ensure viability for subsequent characterization and analysis. All microorganisms were stained with a 0.02% Sudan Black solution to assess their PHA production capacity. According to Kumar et al. [115], colonies that produced PHA exhibited a distinct dark blue coloration, while non-producing colonies retained their original color. This staining method effectively indicates PHA accumulation in microbial cells, facilitating the differentiation between PHA-producing and non-producing strains. Such differentiation is essential for selecting microorganisms for further investigation and potential applications in bioplastics and sustainable material production.

Identifying PHA-producing pure cultures involved systematically evaluating the isolated microorganisms, encompassing both bacterial and fungal species. Initially, the cultures were assessed based on their growth characteristics on agar plates, with particular attention to colony morphology, including variations in appearance, coloration, and shape [116]. This qualitative assessment facilitated preliminary identification is based on established taxonomic criteria. Subsequently, the cellular morphology of the isolates was analyzed using a light microscope (KERN OBE 134, KERN, and SOHN GmbH, Balingen, Germany). This microscopic examination provided essential information regarding the structural characteristics, such as cell shape, arrangement, and any distinctive features, which are critical for accurate microbial classification. For bacteria, identification followed the procedures outlined in the Manual of Determinative Bacteriology, which included techniques such as Gram staining and the KOH test [116]. Additional tests included oxidase, catalase, and nitrate-reductase assessments [116], along with a series of biochemical tests known as API (Analytical Profile Index, BioMérieux^®^, Lyon, France), utilizing API CH 50, API 20NE, API 20E, API Staph, and API Strep tests. Yeasts were identified using the API 20C AUX test. The final step of the identification of bacteria was a matrix-assisted laser desorption/ionization time of flight mass spectrometry (Microflex LT MALDI-TOF MS, Bruker Daltonics, Bremen, Germany) analysis, which is based on the protein identification of pulsed single ionic analytes (pure microbial culture), coupled with a TOF measuring mass analyzer, and the exact protein mass was determined.

### 2.3. PHA Production from Agricultural Waste via Solid-State Fermentation with Mixed Microbial Cultures

**Figure 1:**
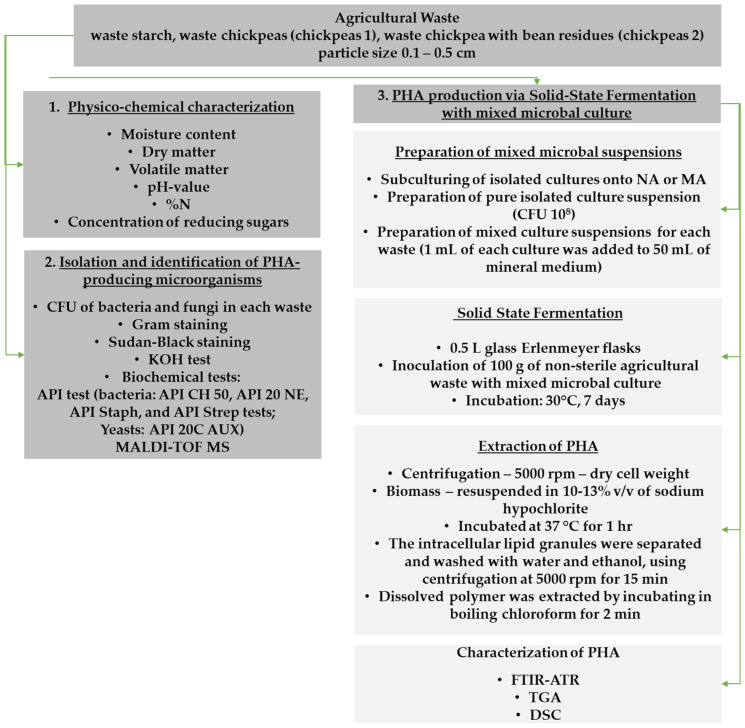
Experimental workflow for PHA production.

Isolated pure cultures capable of producing PHA were prepared as inoculum for PHA production from agricultural waste (Figure 1). Bacterial cultures were cultivated on NA at 37 °C for 24 h, while fungal cultures were cultivated on MA at 28 °C for 48 h. After incubation, each bacterium and fungi were harvested using a sterile inoculation loop and transferred into 25 mL of nutrient broth and malt broth, respectively. These suspensions were then incubated aerobically on a rotary shaker (Biosan PSU-20i Orbital Shaker, Riga, Latvia) at 160 rpm and 30 °C for 18 h to promote further growth and enhance the inoculum for subsequent applications in PHA production. The CFU value of the obtained pure bacterial and fungal suspensions was between 10^8^ and 10^9^. The next step involved preparing three distinct mixed culture suspensions using microorganisms isolated from waste starch, chickpeas 1, and chickpeas 2. One milliliter of each pure culture suspension was added to 50 mL of sterile water and thoroughly homogenized. The obtained suspensions were uniformly applied to 100 g of waste starch, chickpeas 1, and chickpeas 2, respectively. Solid-state fermentation was conducted in 0.5 L glass Erlenmeyer flasks that had been sterilized by autoclaving at 121 °C for 20 min. Each flask was filled with 100 ± 1 g of the inoculated substrate and incubated in a thermostat at 30 °C for 7 days. The initial experimental conditions are summarized in Table 2. At the end of experiments, physical-chemical characterization was also performed (see Section 2.1).

The biomass produced during solid-state fermentation was quantified gravimetrically following two consecutive solid–liquid extractions, as described by Martínez-Avila et al. [66]. In summary, the extraction involved mixing the fermented sample (5–8 g) with distilled water at a 1:3 ratio in an orbital shaker set at 160 rpm and 30 °C for 30 min. The supernatant was then vacuum filtered through AP25 paper and collected quantitatively in Falcon tubes. The filtered solution was centrifuged at 5000 rpm and 4 °C for 15 min, after which the supernatant was discarded. The pellet containing the biomass was washed with distilled water and centrifuged at 5000 rpm for 15 min to recover the solid fraction. The resulting pellet was dried at 60 °C for 24 h and weighed to determine the biomass or cell dry weight (CDW) in the solid sample. PHA extraction from the dried biomass was performed using the method outlined by Law and Slepecky [117,118]. The biomass was resuspended in a 10–13% *v*/*v* sodium hypochlorite solution and incubated at 37 °C for 1 h to disrupt the cell membranes. Intracellular lipid granules were then separated and washed with water, acetone, and ethanol, respectively, through centrifugation at 5000 rpm for 15 min. Finally, the dissolved polymer was extracted by incubating the mixture in boiling chloroform for 2 min, followed by filtration of the solution containing the dissolved polymer. PHA accumulation was calculated according to the following equation, Equation (1) [116]:

(1) $$PHA\;accumulation\left(\%\right)=\frac{Dry\;weight\;of\;extracted\;PHA\;(\frac{g}{L})}{DCW\;(\frac{g}{L})}\times 100\%$$

where DCW is dry weight of biomass.

The characterization of PHA produced after 7 days of experimentation was performed using an FTIR spectrometer (FTIR-8400S, Shimadzu, Kyoto, Japan) equipped with an ATR sampling accessory (MIRacle™ Single Reflection ATR, PIKE Technologies, Fitchburg, WI, USA), covering a spectral range from 4000 to 650 cm^−1^. Approximately 15 mg of the extracted PHA sample was carefully placed on the ATR prism, ensuring complete surface coverage for optimal spectral acquisition. The recorded spectra were then analyzed and processed using IR Solution software 1.6 (Shimadzu, Kyoto, Japan) to accurately identify the characteristic functional groups present in the sample. Each sample was measured at least four times and only the most representative spectra were selected. The thermal properties of PHA, specifically the melting temperature (*T*_m_) and crystallization temperature (*T*_c_), were assessed using differential scanning calorimetry (DSC) on a Mettler Toledo DSC 3 Star System (Mettler Toledo, Columbus, OH, USA). The DSC analysis involved heating samples from room temperature to 200 °C, followed by a three-minute isothermal hold. The samples were then cooled from 200 to −60 °C and reheated to 200 °C. This process generated a thermogram that illustrates the relationship between heat flow and temperature. Additionally, thermogravimetric analysis (TGA) was conducted using a TGA Q500 thermogravimetric analyzer (TA Instruments, New Castle, DE, USA). Approximately 5–7 mg samples were heated from 40 to 700 °C at a constant rate of 10 °C min^−1^ under a nitrogen atmosphere (60 mL min^−1^). The resulting thermograms were analyzed to determine mass loss, thermal stability, and decomposition temperature ranges of the PHA samples.

All experiments and measurements were performed in triplicate. The standard deviation (σ) was calculated using Equation (2):

(2) $$\sigma =\surd \frac{\sum {\left(x-\bar{x}\right)}^{2}}{n}$$

where *x* represents individual measurements, $\bar{x}$ is the mean value, and *n* is the number of measurements. The standard deviation was then expressed as ± and added to the reported results. All calculations were performed using Microsoft Excel (part of the Microsoft Office Suite).

## 3. Results and Discussion

### 3.1. Isolation and Identification of PHA-Producing Microorganisms

**Figure 2:**
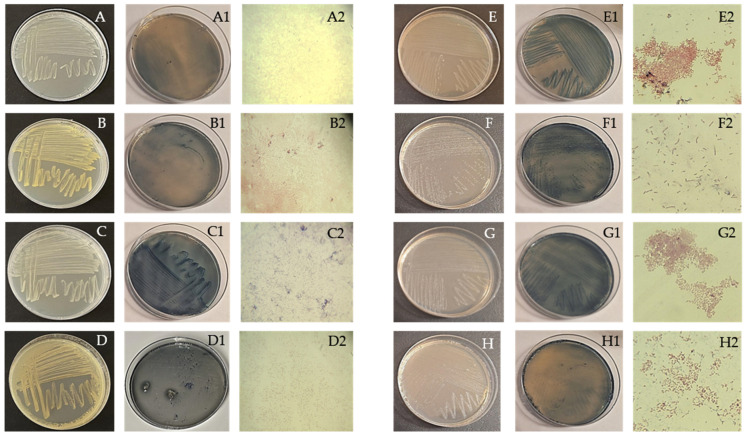
Obtained pure cultures by streaking method, cultures stained with Sudan Black dye, and microphotographs of Gram staining of bacteria isolates *Brevibacillus* sp. (**A**,**A1**,**A2**), *Empedobacter brevis*; (**B**,**B1**,**B2**), *Aneurinibacillus aneurinilyticus*; (**C**,**C1**,**C2**), *Micrococcus* spp.; (**D**,**D1**,**D2**), *Leuconostoc* sp; (**E**,**E1**,**E2**), *Bacillus licheniformis*; (**F**,**F1**,**F2**), *Staphylococcus lentus*; (**G**,**G1**,**G2**), *Citrobacter freundii*; (**H**,**H1**,**H2**), M = 1000×.

Microorganisms with the potential to produce PHA were successfully isolated from three distinct carbon sources: starch, chickpea 1, and chickpea 2. A total of 12 microorganisms were isolated and identified, comprising 8 bacterial cultures and 4 yeast cultures. To assess their capability to produce PHA, the Sudan Black staining method was employed. This technique involves the use of a 0.02% Sudan Black solution [119], which selectively stains intracellular lipid granules, including PHA (Figure 2). The analysis revealed that the colonies exhibited a dark blue coloration, indicating the accumulation of PHA within the cells. This distinct color change serves as a visual marker for PHA production, allowing for easy differentiation between PHA-producing and non-producing strains. Notably, the intensity of the blue coloration correlates with the quantity of PHA accumulated, providing a qualitative assessment of the production capacity of each isolated microorganism. Such visual indicators are critical for selecting optimal microorganisms for further study and potential industrial applications in bioplastics.

Table 3 presents the microorganisms identified from the three agricultural waste sources, accompanied by their morphological characteristics. The successful isolation and characterization of these microorganisms not only emphasize their potential for biopolymer production but also demonstrates the feasibility of leveraging diverse agricultural waste for sustainable PHA synthesis. Notably, the results indicated that most PHA-producing microorganisms were isolated from starch, suggesting that starch serves as a particularly advantageous carbon source for the growth and metabolism of these organisms. This observation can be attributed to several interrelated factors. Starch, as a polysaccharide composed of glucose units, can be easily metabolized by a wide range of microorganisms. The availability of these simple sugars provides an efficient energy source, facilitating microbial growth. Additionally, some microorganisms may possess specific enzymatic pathways, such as amylase, which enable them to efficiently degrade starch into simpler sugars that can be utilized for growth and biopolymer production [120]. Furthermore, the conditions during the isolation and cultivation processes may have favored the growth of starch-utilizing microorganisms over those that metabolize chickpeas. Environmental factors such as pH, temperature, and moisture levels play a significant role in the proliferation of microorganisms, especially those involved in starch degradation [121]. Overall, these findings highlight the significance of selecting appropriate carbon sources to optimize microbial growth and biopolymer production in biotechnological applications, reinforcing the role of agricultural waste as a valuable resource in sustainable PHA synthesis.

Bacteria can be categorized into two primary groups based on their morphological features: rod shaped (*bacilli*) and spherical (*cocci*) [122]. The results of the isolation of bacteria from chickpea 1, chickpea 2, and starch showed that rod-shaped bacteria were the most dominant. Among these were *Brevibacillus* sp., *Empedobacter brevis*, and *Aneurinibacillus aneurinilyticus*, isolated from chickpea 1, along with *Bacillus licheniformis* and *Citrobacter freundii*, isolated from starch. Based on the properties of their cell walls, isolated bacteria were classified as either Gram-positive or Gram-negative. 

According to the literature [123], research has shown that most bacteria capable of producing PHA have been found to be Gram-negative. Among the eight isolated bacterial strains, only two—*Empedobacter brevis* and *Citrobacter freundii*—exhibited red staining when examined under a microscope. This observation indicates that these strains are Gram-negative bacteria, as evidenced by their inability to retain the crystal violet stain used in the Gram staining procedure. This characteristic is a fundamental aspect of their cell wall structure, which is typically thinner and surrounded by an outer membrane containing lipopolysaccharides. The remaining 6 bacterial strains, *Brevibacillus* sp., *Aneurinibacillus aneurinilyticus*, *Micrococcus* spp., *Leuconostoc* sp., *Bacillus licheniformis*, and *Staphylococcus lentus*, showed purple staining upon microscopic examination, indicating that they are Gram-positive bacteria. While Gram-negative bacteria are well-known as the main and most researched producers of PHAs, recent studies highlight that certain Gram-positive bacteria can also produce these biopolymers, particularly PHB [124,125]. This ability is due to the presence of specific genes and enzymes, such as *phaC* (PHA synthase) and *phaA* (β-ketothiolase) [126], which are crucial for the PHA biosynthesis process. These enzymes help polymerize hydroxyalkanoic acid monomers into PHA polymers, allowing Gram-positive bacteria to produce and accumulate significant amounts of PHA when grown under suitable conditions. These findings are consistent with previous reports that recognize Gram-positive genera, such as *Bacillus* [125,127,128], *Rhodococcus* [129,130], *Staphylococcus* [131,132], *Corynebacterium* [133], and *Nocardia* [134,135] as efficient PHA producers. Unlike Gram-negative bacteria, which have an outer membrane rich in lipopolysaccharides, Gram-positive bacteria have a thicker peptidoglycan layer in their cell walls. This structural difference has been shown to provide certain advantages in biotechnological applications, including simplified downstream processing due to the absence of endotoxins and lipopolysaccharides, which are often problematic in the purification of biopolymers from Gram-negative strains. Furthermore, Gram-positive bacteria tend to grow faster and are more resistant to various environmental stresses, making them appealing candidates for large-scale industrial applications. Their ability to accumulate PHA under nutrient-limiting conditions, such as nitrogen or phosphorus limitation, coupled with their robustness in diverse environments, positions Gram-positive bacteria as promising microorganisms for sustainable biopolymer production.

Figure 2 illustrates the bacterial strains cultured on NA plates, including those stained with Sudan Black dye, alongside microscopic images of Gram-stained bacteria. The findings from the Gram staining, KOH test, and biochemical assays are summarized in Table 4.

For additional confirmation of Gram staining results, a rapid KOH test was also conducted. Since Gram-negative bacteria have a thin peptidoglycan layer in their cell walls, in contact with 3% KOH solution they form thin filaments, indicating a positive KOH test result (+). On the other hand, Gram-positive bacteria do not form filaments with KOH solution, which is marked as a negative KOH test result (−). When comparing the results of Gram staining and KOH test (Table 4), a few inconsistencies from expected outcomes were noticed, particularly for *Brevibacillus* sp., *Aneurinibacillus aneurinilyticus*, *Micrococcus* spp., and *Citrobacter freundii*. Several factors can lead to false results in the KOH test. In the case of Gram-negative bacteria, inaccurate KOH test results may occur due to an insufficient amount of bacteria. In contrast, for Gram-positive bacteria, false results tend to occur due to an excessive number of bacteria or growth of bacterial colonies with “sticky” structure [136].

A crucial step for identification of bacteria is conducting biochemical tests, as these tests reveal the presence of specific enzymes, such as oxidase, catalase, and nitrate-reductase [137]. Catalase is produced by many aerobic and most facultative anaerobic bacteria, breaking down H_2_O_2_ into water and oxygen [138]. The catalase test detects this enzyme, with a positive result shown by oxygen bubbles. Aerobic bacteria often contain cytochrome C oxidase, which reduces oxygen to water. Tests using *N*,*N*,*N*’,*N*’–tetramethyl-*p*-phenylenediamine turn purple in its presence [138]. The final biochemical test identifies nitrate-reducing bacteria by detecting their ability to reduce nitrate to nitrite, indicated by the appearance of a pink color [139]. All the isolated bacteria, with the exception of *Leuconostoc* sp., showed positive results in catalase test (Table 4). In this study, various API strip tests were utilized to identify and characterize isolated bacterial strains, leveraging their metabolic and biochemical profiles. These standardized kits, including API CH50, API 20E, API 20NE, API Staph, and API Strep for bacteria, and API 20C AUX [140] for yeasts, provide efficient, reliable, and reproducible tools for species identification in microbiological diagnostics. Each test uses specific reagents and interprets results through a comprehensive database, enabling precise species classification. The API CH50 strip [141] is designed to test the fermentation of 50 different carbohydrates and assess related metabolic activities. This system is particularly effective for classifying both Gram-positive and Gram-negative bacteria based on their ability to metabolize various compounds, making it a valuable tool for identifying bacteria from diverse environmental and clinical samples. In this study, API CH50 was used for the identification of *Brevibacillus* sp., *Aneurinibacillus aneurinilyticus*, *Leuconostoc* sp., and *Bacillus licheniformis*. For the identification of *Brevibacillus* sp., *Bacillus licheniformis* and *Aneurinibacillus* aneurinilyticus API 20NE [142] also used *s* for broader metabolic characterization, including oxidase, nitrate reduction, and enzymatic activities. The API 20NE strip evaluates 20 biochemical reactions, including carbohydrate assimilation, and is particularly useful for identifying opportunistic pathogens outside the Enterobacteriaceae family. It was used for identifying *Empedobacter brevis*. API Staph [143] identifies *Staphylococcus* species and other Gram-positive cocci by analyzing traits like enzyme activity (e.g., coagulase and urease) and carbohydrate fermentation. This strip was used for identifying *Micrococcus* spp. and *Staphylococcus lentus*. The API Strep test [144], tailored for identifying *Streptococcus* species, tests for their ability to ferment sugars and perform other specific metabolic activities. However, no strains were identified using this system in this study. Finally, *Citrobacter freundii*, a glucose-fermenting Gram-negative bacterium from the Enterobacteriaceae family, was identified using the API 20E kit [145]. This test is specifically designed for Enterobacteriaceae, assessing characteristics like citrate utilization, lysine decarboxylase activity, and fermentation patterns. The identification process included the use of MALDI-TOF MS, which was employed as the final confirmation step following the API strip tests [146]. This technique is based on protein profiling, providing high-resolution identification of microorganisms through mass spectrometric analysis. MALDI-TOF MS identifies microorganisms by analyzing the protein fingerprint of pure microbial cultures. It uses a laser to ionize the sample, producing singly charged ions from the proteins present. These ions are then measured by a time-of-flight mass analyzer, which determines the exact mass of the proteins. By comparing the resulting spectra to a comprehensive database, the genera and species of the isolates were accurately confirmed. This method offers advantages such as rapid identification, high specificity, and reproducibility, making it an essential complement to biochemical testing for microbial classification.

Among the identified bacteria, only a few of them have been previously explored and reported for PHA production. According to the literature, *Aneurinibacillus* sp. [147], *Citrobacter freundii*, and *Leuconostoc* spp. [148] are known to produce PHB. Lastly, bacteria from genera *Micrococcus* and *Staphylococcus* are associated mostly with scl-PHA production [147].

**Figure 3:**
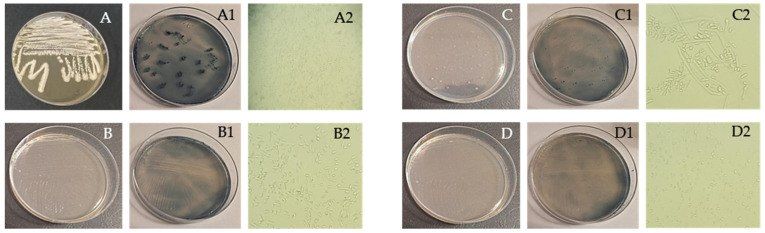
Obtained pure cultures by streaking method, cultures stained with Sudan Black dye, and microphotographs of yeast isolates *Trichosporon asahii* (**A**,**A1**,**A2**), *Cryptococcus humicola*; (**B**,**B1**,**B2**), *Geotrichum klebahnii*; (**C**,**C1**,**C2**), *Candida krusei*; (**D**,**D1**,**D2**), M = 400×.

Studies on yeast-based PHA production are less extensive than those focused on bacteria. However, in this investigation, four yeast strains were identified, and their morphological characteristics were examined using a light microscope, following the methodologies outlined in “Introduction to Industrial Mycology” [149]. Table 3 provides detailed information on these characteristics. Figure 3A–D shows yeasts cultured on MA plates, including strains stained with Sudan Black dye, along with microscopic images of the yeasts. *Candida krusei* stands out as the only yeast species with documented evidence of PHA production. Studies have shown that this yeast can produce the polymer PHB, with accumulation levels ranging from 2.44% to 9.26% [150].

### 3.2. PHA Production from Agricultural Waste via Solid-State Fermentation with Mixed Microbial Cultures

To optimize SSF implementation, a comprehensive physico-chemical analysis was undertaken to assess the fermentation suitability of three substrates. This analysis included determination of MC, DM, VM, pH, RS concentration, and CFU (Table 2 and Table 5). According to the initial conditions (Table 2), the moisture content of the substrates ranged from 44% to 60%, reflecting the inherent characteristics of each material [151]. The literature indicates that an MC range of 40–70% is generally optimal for bacterial and yeast growth [152]. Among the substrates, chickpea 2 exhibited the highest MC at 59.86%, while starch showed the lowest at 44.77%. All substrates had high VM, between 97% and 99%. Initial pH values of the substrates were within a range of 4.450 to 5.550, consistent with reported values in the literature [153,154]. Chickpea 2 had the highest concentration of reducing sugars, at 56.78 mg/g_DM_. 

At the end of the SSF process (Table 5), it was observed that the moisture content (MC) increased in both chickpea 1 and chickpea 2. The changes in volatile matter were minimal across all three substrates (3–6%), suggesting that while some components of the substrate were metabolized, the overall matrix remained intact. In contrast, a significant reduction in the concentrations of RS was observed in chickpea 2 from the beginning to the end of fermentation (Table 2 and Table 5). This decline suggests that microorganisms actively utilized the available RS during fermentation, resulting in their depletion. This observation aligns with findings reported in the literature, which highlight the metabolic activity of microorganisms in converting available sugars into fermentation by-products. [155,156]. The observed differences in the changes in RS and volatile matter (VM) can be explained by the distinct metabolic pathways employed by the microorganisms. RS, being readily available, is preferentially consumed by microorganisms for energy and growth, leading to its reduction. In contrast, volatile matter consists of more complex organic compounds, such as cellulose, lignin, and hemicellulose, which are not immediately available for microbial utilization. The complex hierarchical structure of cellulose, consisting of crystalline nanofibrils intertwined with lignin and hemicellulose, presents a barrier to microbial and enzymatic degradation, making the breakdown of cellulose into fermentable sugars challenging [157,158]. This intricate structure highlights the difficulty in developing universal pretreatment strategies for efficient biomass conversion. By implementing effective pretreatment strategies, such as dilute acid pretreatment, it is possible to optimize the substrate composition and enhance the bioavailability of carbon sources, thereby maximizing the potential for microbial growth and biopolymer production [158].

The change in CFU during SSF is significantly influenced by the physico-chemical characteristics of solid substrates and its interaction with nutrients and water [153]. The initial values of CFU for bacteria (Table 4) in all substrates were very high. A similar trend can be seen for fungi, although the CFU values were lower. After 7 days of experiment (Table 5), an increase in CFU values of bacteria and fungi was noticed on NA and MA, respectively. This proliferation reflects the microorganisms’ adaptation to the solid substrates and their effective utilization of available nutrients. Notably, the most significant increase in CFU value was observed with chickpea 2, where the CFU on NA increased from 1.2 × 10^9^ to 4.7 × 10^10^ cell/g_DM_.

The final step of SSF involved drying the substrates to determine the dry cell weight (DCW) and extracting the PHA. This extraction used a biphasic system, where chloroform, the lower phase, was used to isolate the PHA [159]. This method relies on the fact that PHA is soluble in organic solvents like chloroform, which allows for the efficient separation of the polymer from the biomass. The results of the PHA extraction from various substrates, summarized in Table 6, indicated relatively low percentages of PHA accumulation overall. However, chickpea 2, which exhibited the highest CFU value (Table 5), demonstrated a notably higher accumulation percentage of 13.81%. In contrast to chickpea 1 and starch, which yielded a greater diversity of microbial cultures, only two distinct microbial cultures were isolated from chickpea 2. The observed limited microbial diversity in cultures isolated from chickpea 2 may have reduced competition among microorganisms, thereby enhancing their metabolic efficiency and promoting greater accumulation of PHAs. This observation is consistent with studies [160,161,162] showing that lower microbial diversity can improve metabolic output by reducing interspecies competition for resources. With fewer species present, microorganisms can more effectively utilize available carbon sources for PHA production. Additionally, the concentration of microbial cultures can influence biopolymer accumulation, as reduced competition allows for optimized resource utilization and enhanced microbial growth. In a previous study, *Plasticicumulans* was enriched with 27.6% and 50.6% as the most abundant populations, under high and low feast dissolved oxygen (DO) conditions, respectively. When both cultures were fed with a synthetic mixture of the four volatile fatty acids for PHA accumulation, butyrate and valerate were always taken up first, followed by acetate and propionate, regardless of the DO levels applied. It seemed that the abundance of *Plasticicumulans* in an enriched mixed microbial culture for PHA accumulation had a direct impact on substrate competition, with a clear preference for butyrate and valerate over acetate and propionate [162]. It is noteworthy that chickpea 2 contained a strain of *Micrococcus*, which previous studies have associated with the production of scl-PHAs [147]. This suggests that the presence of *Micrococcus* in chickpea 2 may have played a pivotal role in the biosynthesis of PHAs, thereby contributing to the higher accumulation observed in this substrate (Table 6). The implications of microbial diversity and strain-specific capabilities underscore the importance of substrate selection in optimizing PHA production during fermentation processes. This finding underscores the suitability of chickpea 2 as a substrate for microbial growth and PHA production, highlighting its potential for optimizing biopolymer yields. The correlation between CFU values and PHA accumulation can be attributed to several factors. First, the robust microbial population indicated by the CFU values suggests that chickpea 2 provided an optimal environment for microbial proliferation, which in turn facilitated higher PHA biosynthesis. Furthermore, the nutritional composition of chickpea 2 may have favored the metabolic pathways leading to PHA production, thereby enhancing the accumulation of this biopolymer [163]. Overall, the results emphasize the importance of substrate selection in SSF processes for PHA production, as the characteristics of the substrate directly influence both microbial growth and the efficiency of PHA accumulation [160,164]. Moreover, the choice of substrate can reduce or intensify competition among microbial communities, further influencing the efficiency of PHA synthesis [164]. Future investigations could focus on refining the extraction techniques and optimizing fermentation conditions to further improve PHA yields from different substrates. Additionally, incorporating preprocessing steps for agricultural waste prior to fermentation may enhance the accessibility of carbon sources for microorganisms. This could involve methods such as physical treatment (e.g., grinding or milling), chemical treatment (e.g., acid or alkali hydrolysis), or biological pretreatment (e.g., enzymatic degradation) [165]. By breaking down complex molecules into simpler sugars and increasing the surface area of the substrates, preprocessing could facilitate more efficient microbial metabolism and higher PHA production rates. Such approaches not only optimize the fermentation process but also contribute to the overall sustainability of using agricultural waste for biopolymer synthesis.

### 3.3. Characterization of Extracted PHA

#### 3.3.1. FTIR-ATR Spectroscopy Analysis of the Extract

**Figure 4:**
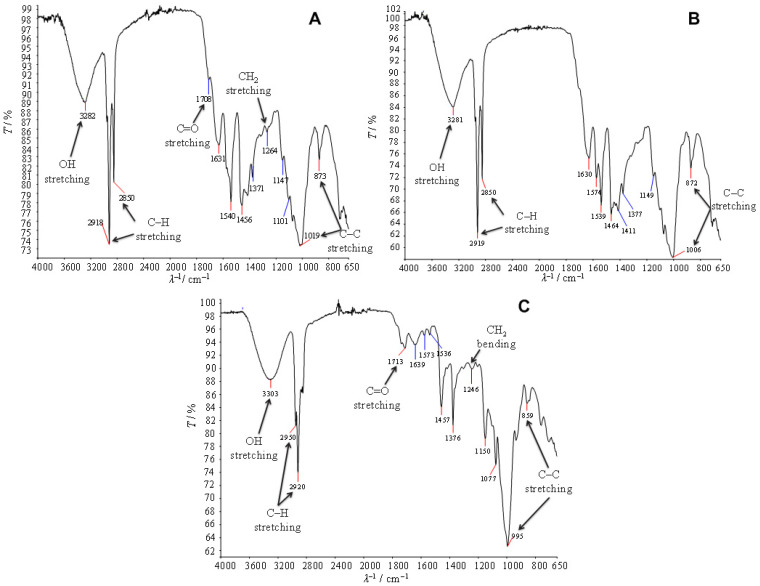
FTIR spectra of PHAs obtained in the fermentation processes using (**A**) chickpea 1; (**B**) chickpea 2; and (**C**) starch as a substrate.

**Figure 5:**
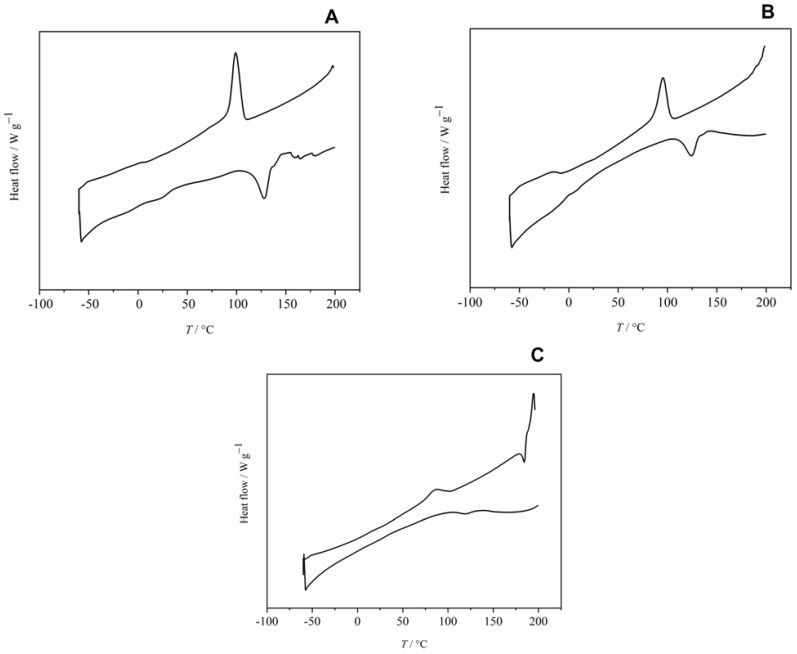
DSC spectra of extracted samples (PHA) obtained in the fermentation processes using (**A**) chickpea 1; (**B**) chickpea 2; and (**C**) starch as a substrate.

In FTIR spectroscopy, several characteristic functional groups and their corresponding absorption peaks are associated with PHAs. These groups are crucial for confirming the presence and structure of PHAs in a sample. All infrared (IR) spectra (Figure 4) exhibit a broad peak between 3281 and 3303 cm^−1^, indicating the presence of hydroxyl groups (–OH) and potentially water, which complicates the definitive identification of PHAs [166]. In Figure 5A, the IR spectrum for chickpea 1 shows absorption peaks at 2918 and 2850 cm^−1^, corresponding to asymmetric and symmetric stretching of methyl (–CH₃) and methylene (–CH₂) groups. The peak at 1708 cm^−1^ signifies C=O stretching associated with carbonyl bonds, while peaks at 1264, 1371, and 1456 cm^−1^ represent bending vibrations of methyl and methylene bonds. Additionally, absorption peaks at 1019 and 873 cm^−1^ indicate the presence of C–C bonds. Figure 5B presents the IR spectrum for chickpea 2, which closely resembles that of chickpea 1, with notable variations in the methyl and methylene stretching peaks at 2919 and 2850 cm^−1^, as well as C–C bond stretching peaks at 1006 and 872 cm^−1^. The IR spectrum for starch (Figure 5) also reveals characteristic peaks associated with PHA structure. Peaks at 2950 and 2920 cm^−1^ indicate asymmetric stretching of methyl and methylene groups, while smaller peaks at 1713 and 1246 cm^−1^ confirm carbonyl bond stretching and bending of methyl and methylene bonds, respectively [167]. Overall, the IR spectral analysis reinforces the identification of PHB in the substrates, emphasizing the molecular characteristics common to PHA structures. Further investigation may be necessary to clarify the presence of water and other components that could affect PHA quantification.

#### 3.3.2. DSC Analysis of the Extract

The DSC thermograms of extracted samples derived from chickpea 1 and starch exhibit a broad endothermic peak during the first heating cycle, indicative of water evaporation from the structural matrix of the samples. This peak is absent in the thermogram of the extracted samples obtained from chickpea 2. All thermograms display irregular profiles characterized by numerous sharp peaks, which provide critical information regarding the glass transition temperature (*T*_g_), melting temperature (*T*_m_), crystallization temperature (*T*_c_), and the enthalpies of melting and crystallization (Δ*H*_m_ and Δ*H*_c_, respectively). Such thermal data are essential for the characterization and identification of the synthesized PHA. The detailed results of the DSC analysis are summarized in Table 7.

The cooling cycle thermograms reveal a prominent exothermic peak, indicating the crystallization of PHA. This peak is distinctly observed in the thermograms (Figure 5A,B) for PHA samples derived from both chickpea 1 and chickpea 2, with crystallization temperatures recorded at 99.10 °C and 95.24 °C, respectively. These findings align with the published literature [168], which reports similar crystallization temperatures for polyhydroxybutyrate-co-valerate (PHBV), exhibiting a crystallization temperature of approximately 70 °C for copolymers containing 3, 8, and 10 mol% of hydroxyvalerate (HV). In contrast, copolymers with higher HV content, specifically 17% and 30%, demonstrated lower crystallization temperatures of 60 °C and 40 °C, respectively. These comparisons underscore the thermodynamic behavior of PHAs and highlight the influence of compositional variations on their crystallization properties. The observed crystallization temperatures in the current study suggest that the molecular arrangement and interaction of the components in the PHA samples significantly affect their thermal properties. The melting curve, specifically the second heating cycle curve of PHA obtained from chickpea 2 (Figure 5B), reveals a glass transition temperature of 1.81 °C. Further analysis of the melting curve demonstrates the presence of distinct endothermic peaks, with melting temperatures of 128.16 °C, 124.52 °C, and 119.53 °C corresponding to the PHAs extracted from chickpea 1, chickpea 2, and starch, respectively. These melting temperatures align closely with those reported by Dias et al. [169], which indicate melting point 133 °C PHBV containing 25 mol% of hydroxyvalerate (HV), as well as for the copolymer P(3HB-co-6 mol% 3HA). The variations in melting temperature suggest that the composition of the substrates influences the thermal properties of the resulting PHA. The higher crystallization and melting temperatures observed in this study may be indicative of a more crystalline structure, which could contribute to improved mechanical properties and thermal stability of the produced biopolymers. This ordered structure may also reflect the specific microbial metabolic pathways utilized during PHA production, which can affect the copolymer composition and consequently its thermal characteristics. Additionally, the crystallization behavior of PHAs is crucial for their processing and application in biodegradable materials. Understanding these thermal properties helps in optimizing the production and processing conditions to enhance the performance of PHAs in various applications, including packaging, agricultural films, and other environmentally friendly products. Further investigation into the relationship between the composition of the substrate and the resulting thermal properties of PHA could provide insights for optimizing biopolymer production from agricultural waste.

#### 3.3.3. TGA of the Extract

**Figure 6:**
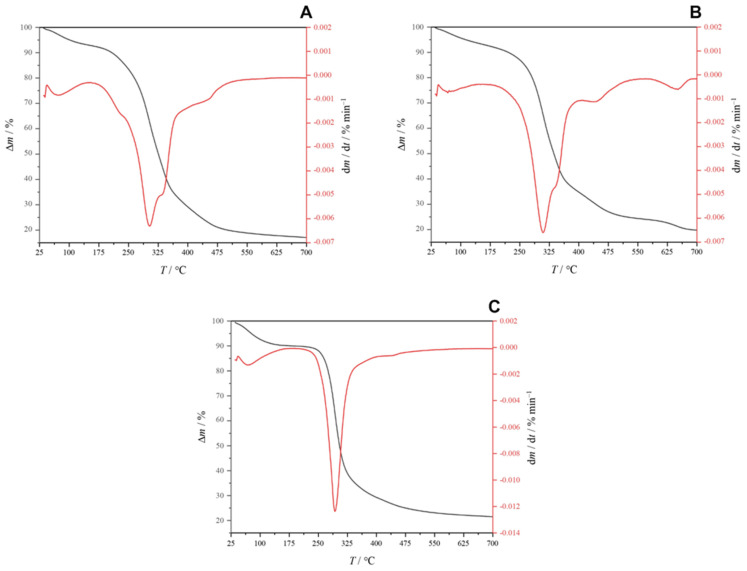
Thermograms of PHAs obtained in the fermentation processes using (**A**) chickpea 1; (**B**) chickpea 2; and (**C**) starch as a substrate.

The TGA curves (Figure 6) obtained after seven days of SSF indicate a single-stage thermal degradation process for PHA, consistent with findings by Apiwatanapiwat et al. [129] and Akbari et al. [170]. This single-stage degradation suggests a uniform thermal behavior of the obtained PHA in this study, which may be attributed to the specific microbial activity and substrate conditions during fermentation. In contrast, Dikshit et al. [171] reported a two-stage thermal degradation profile for PHB synthesized using *Cupriavidus necator* and *Bacillus megaterium*, with degradation temperatures ranging from 100 °C to 160 °C. They attributed this phenomenon to the selective evaporation of adsorbed solvents, such as chloroform, which may influence the thermal properties of the synthesized PHB. The presence of these solvents can lead to variations in the degradation mechanisms, highlighting the importance of synthesis conditions on the thermal stability of biopolymers. The highest mass loss, recorded at 71.64%, was observed in the PHA sample derived from chickpea 1. This significant mass loss may reflect the degradation of less thermally stable components or impurities within the sample. The maximum degradation temperatures (*T*_d,max_) for the PHAs obtained in this study were found to be 302.75 °C, 309.06 °C, and 293.37 °C for samples from chickpea 1, chickpea 2, and starch, respectively. These temperatures align with those reported by Dikshit et al. [170], reinforcing the expected degradation profile for PHB. Notably, the *T*_d,max_ values observed in this study are higher than those reported for commercial PHB, which exhibit degradation temperatures of 415 °C and 289 °C. This indicates that the PHB synthesized in this research is thermally more resistant than the commercially available variants. The enhanced thermal stability of the produced PHB may be advantageous for applications requiring materials that can withstand higher temperatures without degradation, further emphasizing the potential of utilizing agricultural waste as a substrate for biopolymer production.

## 4. Conclusions

This study explores the potential of utilizing agro-industrial waste, specifically starch and chickpea byproducts, as substrates for PHA production. A total of eight bacterial and four yeast strains capable of PHA biosynthesis were successfully isolated from these waste materials. Comprehensive characterization of the substrates, both before and after SSF, provided critical data on moisture content, dry matter, volatile matter, nitrogen and carbon content, and reducing sugar levels. The SSF process, conducted over seven days, achieved a maximum PHA accumulation of 13.81% from the chickpea 2 substrate. Structural and thermal analyses using FTIR-ATR, TGA, and DSC indicated the synthesis of PHB. While the results demonstrate the feasibility of PHA production from agro-industrial waste, further research is required to enhance yield, particularly through substrate pretreatment, optimization of fermentation conditions, and exploration of alternative substrates. Additionally, more detailed structural characterization will facilitate the development of bioplastics with tailored properties. This study contributes to advancing sustainable PHA production, supporting both environmental sustainability and the principles of the circular economy.

## Figures and Tables

**Table 1:** Agro-industrial waste type and microorganisms used for production of PHA.

Agro-Industrial Waste	Pretreatment Method	Microorganism	PHA Yield	Ref.
Apple pulp waste	n.a.	*Pseudomonas citronellolis* NRRL B-2504	30 ± 1.7%	[76]
Sugar beetroot pulp	Enzymatic–recombinant endoglucanase (rCKT3eng), chemical hydrolysis	*Haloarcula* sp. TG1	45.6%/17.8%	[77]
Wheat grains	Acidic hydrolysis (hydrochloric acid)	*Bacillus* sp. NII2	1.413 mg/L	[78]
Tequila bagasse	Bacterial pretreatment (*Saccharophagus degradans* ATCC 43961)	*Saccharophagus degradans* ATCC 43961	1.5 mg/L	[79]
Coffee ground	*n*-hexane oil extraction, acidic hydrolysis (sulphuric acid), and enzymatic digestion	*Burkholderia cepacia* ATCC 17759	2.69 ± 0.07 g/L	[80]
Canola oil	n.a.	*Wautersia eutropha* ATCC 17699	18.27 g/L	[81]
Sugarcane molasse	n.a.	*Bacillus cereus* SPV	61.07%	[15]

**Table 2:** Physico-chemical and microbiological properties of agricultural wastes (standard deviation was between ±0.021 and ±0.044).

Substrate	*w*(MC)/%	*w*(DM)/%	*w*(VM)/%	pH/−	*ω*(RS)/mg g_DM_^−1^	CFU_bacteria_/g_DM_	CFU_fungi_/g_DM_	*w*(C)/%	*w*(N)/%
Chickpea 1	58.06	41.94	97.69	5.545	45.47	9.8 × 10^9^	1.6 × 10^7^	54.27	3.93
Chickpea 2	59.86	40.14	97.72	5.011	56.78	1.2 × 10^9^	1.5 × 10^8^	54.28	4.02
Starch	44.77	55.23	99.36	4.453	33.54	1.5 × 10^8^	7.4 × 10^6^	55.20	0.02

MC—moisture content; DM—dry matter; VM—volatile matter; RS—reducing sugars; CFU—Colony Forming Unit.

**Table 3:** Morphology of identified microorganisms isolated from agricultural waste.

Substrate	Identified Microorganism	Morphology
Chickpea 1	*Brevibacillus* sp.	Transparent with flat elevation, and regular round configuration, rod shaped
	*Empedobacter brevis*	Orange with flat elevation, and regular round configuration, rod shaped
	*Aneurinibacillus aneurinilyticus*	Brownish with raised elevation, and regular round configuration, rod shaped
Chickpea 2	*Micrococcus* spp.	Orange with flat elevation, and regular round configuration, round shaped (cocci)
	*Trichosporon asahii*	White and cracked in the middle with smooth and shiny edges
Starch	*Leuconostoc* sp.	White with flat elevation, and regular round configuration, cocci/coccobacilli
	*Bacillus licheniformis*	White with raised elevation, wavy and smooth edges, rod shaped
	*Staphylococcus lentus*	Transparent with raised elevation, and regular round configuration, round shaped (cocci)
	*Citrobacter freundii*	Transparent with raised elevation, irregular shape with twisted edges, rod shaped
	*Cryptococcus humicola*	Yellowish with raised elevation, round shape with jagged edges
	*Geotrichum klebahnii*	White with flat elevation, filamentous shape with jagged edges
	*Candida krusei*	White with raised elevation, and regular round configuration

**Table 4:** Results of Gram staining, KOH test, and biochemical tests for bacteria isolates (+ is for Gram positive and − is for Gram negative).

Identified Bacteria	Gram Staining	KOH Test	Oxidase	Catalase	Nitrate-Reductase
*Brevibacillus* sp.	+ve	+	+	+	+
*Empedobacter brevis*	−ve	+	+/−	+	−
*Aneurinibacillus aneurinilyticus*	+ve	+	+	+	−
*Micrococcus* spp.	+ve	+	+/−	+	+/−
*Leuconostoc* sp.	+ve	−	−	-	−
*Bacillus licheniformis*	+ve	−	−	+	+
*Staphylococcus lentus*	+ve	−	−	+	+
*Citrobacter freundii*	−ve	−	−	+	+

**Table 5:** Physico-chemical and microbiological properties of used substrates at the end of experiment (standard deviation was between ±0.012 and ±0.053).

Substrate	*w*(MC)/%	*w*(DM)/%	*w*(VM)/%	pH/-	*ω*(RS)/mg g_DM_^−1^	CFU_bacteria_/g_DM_	CFU_fungi_/g_DM_	*w*(C)/%	*w*(N)/%
Chickpea 1	73.66	26.34	93.99	5.557	30.34	3.8 × 10^10^	2.4 × 10^8^	52.21	1.73
Chickpea 2	74.99	25.01	91.90	5.219	12.36	4.7 × 10^10^	2.1 × 10^8^	51.05	1.10
Starch	45.90	54.10	96.32	7.219	21.04	3.4 × 10^9^	1.3 × 10^8^	53.51	0.07

MC—moisture content; DM—dry matter; VM—volatile matter; RS—reducing sugars; CFU—colony forming unit.

**Table 6:** Percentages of PHA accumulation in mixed microbial cultures using agricultural wastes.

Substrate	PHA Accumulation/%
Chickpea 1	5.42 ± 0.062
Chickpea 2	13.81 ± 0.048
Starch	5.29 ± 0.086

**Table 7:** DSC analysis results for PHA obtained from used substrates after 7 days of SSF.

Sample	*T*_g_/°C	*T*_m_/°C	Δ*H*_m_/J g^−1^	*T*_c_/°C	Δ*H*_c_/J g^−1^
Chickpea 1	/	128.16 ± 0.007	−8.46 ± 0.002	99.10 ± 0.001	16.20 ± 0.009
Chickpea 2	1.81 ± 0.007	124.52 ± 0.010	−6.28 ± 0.017	95.24 ± 0.001	11.33 ± 0.007
Starch	/	119.53 ± 0.007	−0.46 ± 0.021	85.37 ± 0.006	1.96 ± 0.010

*T*_g_—glass transition temperature; *T*_m_—melting temperature; Δ*H*_m_—melting enthalpy; *T*_c_—crystallization temperature; Δ*H*_c_—crystallization enthalpy.

## Data Availability

Data are contained within the article.
